# Full-Length Minor Ampullate Spidroin Gene Sequence

**DOI:** 10.1371/journal.pone.0052293

**Published:** 2012-12-14

**Authors:** Gefei Chen, Xiangqin Liu, Yunlong Zhang, Senzhu Lin, Zijiang Yang, Jan Johansson, Anna Rising, Qing Meng

**Affiliations:** 1 Institute of Biological Sciences and Biotechnology, Donghua University, Shanghai, People's Republic of China; 2 Department of Biochemistry and Molecular Biology, Dalhousie University, Halifax, Nova Scotia, Canada; 3 KI-Alzheimer Disease Research Center, NVS (Neurobiology, Care Sciences, and Society) Department, Karolinska Institutet, Stockholm, Sweden; 4 Department of Anatomy Physiology and Biochemistry, The Biomedical Centre, Swedish University of Agricultural Sciences, Uppsala, Sweden; 5 Institute of Mathematics and Natural Sciences, Tallinn University, Tallinn, Estonia; University of South Florida College of Medicine, United States of America

## Abstract

Spider silk includes seven protein based fibers and glue-like substances produced by glands in the spider's abdomen. Minor ampullate silk is used to make the auxiliary spiral of the orb-web and also for wrapping prey, has a high tensile strength and does not supercontract in water. So far, only partial cDNA sequences have been obtained for minor ampullate spidroins (MiSps). Here we describe the first MiSp full-length gene sequence from the spider species *Araneus ventricosus*, using a multidimensional PCR approach. Comparative analysis of the sequence reveals regulatory elements, as well as unique spidroin gene and protein architecture including the presence of an unusually large intron. The spliced full-length transcript of MiSp gene is 5440 bp in size and encodes 1766 amino acid residues organized into conserved nonrepetitive N- and C-terminal domains and a central predominantly repetitive region composed of four units that are iterated in a non regular manner. The repeats are more conserved within *A. ventricosus* MiSp than compared to repeats from homologous proteins, and are interrupted by two nonrepetitive spacer regions, which have 100% identity even at the nucleotide level.

## Introduction

Orb-weaving spiders of the superfamily Araneoidea use specialized abdominal glands to manufacture up to seven different types of protein-based silks or glues, each of which has specific functions and mechanical properties [1]. These seven different silks and glues have evolved highly variable mechanical properties and biological functions. The major ampullate spidroins (MaSps) make up the dragline silk, that is used as a safety line and for the frame of the web. The fiber displays both high tensile strength and extensibility, and is one of nature's best performing materials [2], [3]. The minor ampullate silk is used for prey wrapping and for the auxiliary spiral that further stabilizes the web, and is similar to dragline silk in tensile strength but has lower elasticity and irreversibly deforms when stretched [3]–[6]. In contrast to dragline silk, minor ampullate silk does not supercontract when hydrated [7], [8]. Flagelliform (Flag), or capture silk is highly elastic and coated with glue, and forms the extremely extensible capture spiral of the orb web [2], [9]. Aciniform, wrapping silk is used for wrapping and immobilizing prey, building sperm webs, for web decorations, and also as an egg case liner [1], [10], [11]. Cylindriform silk, also referred to as tubuliform silk, is only secreted by female spiders during the reproductive season and forms the tough outer shell of the egg case, that is sufficiently robust to protect eggs from a variety of threats, such as predator and parasitoid invasion, temperature fluctuations or aqueous environments [12]–[14]. Pyriform silk forms an attachment disc that lashes together the joints of the web and attaches the dragline to substrates, and is used for prey capture and locomotion [15], [16]. Web glues, secreted from aggregate glands, are used by the spider to coat the spiral threads to help capture prey, and are one of the most effective biological glues known [17], [18].

Spider silk proteins – spidroins – are large and defined by their unique repetitive segments as well as non-repetitive N- and C-terminal regions [11], [15], [19]–[22]. Some spidroins contain short, simple repeat units, whereas others are composed of longer, more complicated repeats [11], [15], [21], [22]. To date, most published spidroin gene or cDNA sequences are not full-length sequences due to lack of the 5′-end of the complete message, probably as a result of that cloning methods are biased to amplification of 3′- regions of mRNAs. Furthermore, hurdles associated with cloning and sequencing long stretches of repetitive DNA or large size transcripts make it difficult to obtain full-length gene or cDNA sequences [23]–[26]. However, there have been a limited number of full-length genomic DNA/cDNA silk sequences reported (Table 1). The full-length genomic DNA sequences for MaSp1 and MaSp2 from *Latrodectus hesperus* [21], and two full-length cDNA silk sequences of cylindriform spidroin (CySp)1 and CySp2 from *Argiope bruennichi* have been described [27]. The limited availability of full-length genomic DNA sequences presents a major obstacle for studies of spidroin gene architecture and regulation, as well as in phylogenetic analyses.

**Table 1:** Characterized full-length spider silk gene sequences.

Spidroin	Species	DNA/cDNA	Full size (kb)	Introns	Database
MaSp1	*Latrodectus hesperus*	DNA	9.4	No	GenBank No. EF595246
MaSp2	*Latrodectus hesperus*	DNA	11.3	No	GenBank No. EF595245
CySp1	*Argiope bruennichi*	cDNA	9.1	Unknown	DDBJ No. AB242144
CySp2	*Argiope bruennichi*	cDNA	9.8	Unknown	DDBJ No. AB242145
MiSp	*Araneus ventricosus*	DNA	10.9	One large	GenBank No. JX513956

Minor ampullate silk may be particularly interesting for biomedical applications since it is strong and does not supercontract in water. Only partial minor ampullate spidroin (MiSp) sequences have been reported to date. Partial MiSp cDNA sequences and genomic restriction mapping from *Nephila clavipes* have detected no introns in the repetitive regions, but identified a part of the repetitive region and the C-terminal region [4]. In this study, we describe the first full-length gene sequence for MiSp, including its flanking non-coding regions, obtained by screening a fosmid genomic library from the orb-weaver *Araneus ventricosus* by a multidimensional PCR method.

**Figure 1:**
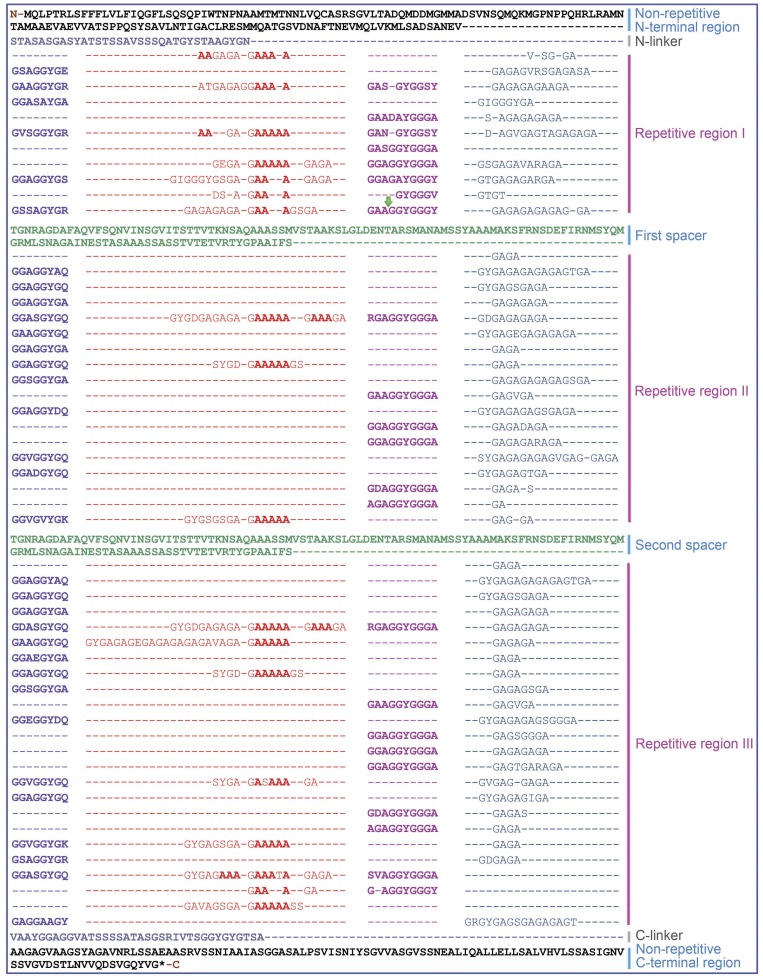
Complete amino acid sequence for *A. ventricosus* minor ampullate spidroin (**MiSp**)**.** The sequence is read from left to right and top to bottom. The first methionine (Met) marks the start position and the asterisk denotes the stop position. The protein is dominated by poly-A (red), GGX (blue and fuchsia), GGGX (fuchsia) and GX (red and cyan) motifs. The protein sequence can be divided into nonrepetitive N- and C-terminal regions and a predominantly repetitive region. The repetitive region is interrupted by two nonrepetitive spacer regions which have 100% identity. Gaps (–) have been inserted in order to align repeat units within a type. Non-repetitive N- and C-terminal regions are in black, spacers are in green and linker regions are in gray. The green arrow marks the intron insertion position.

**Table 2:** Primers used for intron identification.

Primer	Sequence(5′–3′)
Out-S	AGGCGCAAGAGGAGCGGATAGTG
Out-F(RT primer)	GATGTTATCGAATGCCGGTGCGA
In-S	CAGCTGGATACGGTGGAGGAGTTG
In-F	TTATGCTGCTGCTATGGCGAAATC

## Materials and Methods

### Fosmid genomic library construction and screening

*A. ventricosus* individuals were collected in Shanghai, China, frozen in liquid nitrogen, and stored at −80°C. No specific permits were required for the described field studies; the location from which the spiders were collected is not privately owned or protected in any way, and the spiders collected are not endangered or protected. High molecular weight genomic DNA (HMW-gDNA) was isolated by an improved cetyltriethylammonium bromide method from muscle tissues dissected from the cephalothoraxes of ∼20 adult spiders. The HMW-gDNA was sheared mechanically into approximately 40 kb fragments using a 100 μL pipette tip. Subsequently, blunt-end, 5′-phosphorylated DNA was generated using the End-Repair Enzyme Mix (CopyControl™ Fosmid Library Production Kits, Epicentre). Fragments ranging from 25–40 kb were excised from separation gels (avoiding dye staining), purified, and ligated into the vector pCC2FOSTM (Epicentre). The resulting vectors with HMW-gDNAs were packaged using MaxPlaxTM Lambda Packaging Extracts, transfected into EPI300™-T1R T1 phage-resistant *E. coli* cells (Epicentre) and the titers of the phage particles were determined. All operations were performed as described for the CopyControlTM Fosmid Library Production kit (Epicentre). From the generated *A. ventricosus* random genomic Fosmid library, approximately 3.9×105 *E.coli* colonies were picked and arrayed into1026 384-well culture plates (1 clone/well). Each 384-well culture plate was replicated and the original stock plates, containing 8% glycerol, were stored at −80°C.

**Figure 2:**
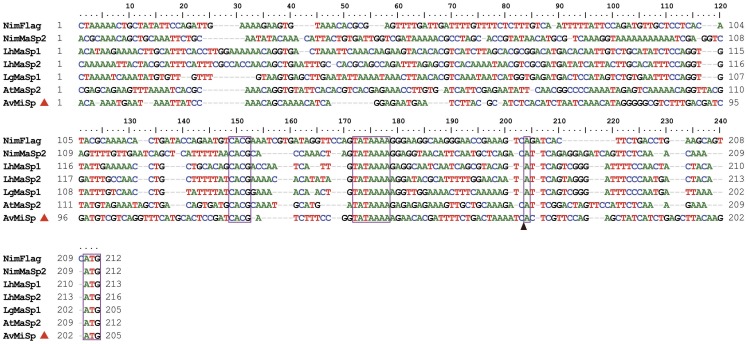
Alignment of genomic DNA upstream sequences. Aligned DNA ranges approximately 210 nucleotides upstream of the predicted transcription start (indicated by a box and a black triangle). The red triangle indicates *A. ventricosus* MiSp upstream sequence. The translation start ATG codons, and the conserved motifs CACG and TATA are boxed. The sequences shown are the following: NimFlag, *N. inaurata madagascariensis* flagelliform (Flag) spidroin gene, upstream (GenBank accession no. AF218623S1); NimMaSp2, *N. inaurata madagascariensis* major ampullate spidroin 2-like gene (GenBank accession no. DQ059135); LhMaSp1, *L. hesperus* major ampullate spidroin 1 (GenBank accession no. EF595246) and 2 (GenBank accession no. EF595245) genes; LgMaSp1, *L. geometricus* major ampullate spidroin 1-like gene (GenBank accession no. DQ059133S1); AtMaSp2, *A. trifasciata* major ampullate spidroin 2 gene (GenBank accession no. DQ059136S1); AvMiSp, *A. ventricosus* minor ampullate spidroin gene (GenBank accession no. JX513956).

For efficient screening of the Fosmid genomic library, a modified method based on 4-dimensional PCR [28] was used. The 1026 384-well culture plates were divided into about 25 sets, each containing about 40 culture plates. Each set of 40 plates was transferred into 40 384-well plates (1 clone/well) which were filled with 60 μL of LB medium containing 12.5 µg/mL chloramphenicol. For each set, the 40 384-well culture plates were arrayed as 5 rows ×8 columns in numerical order. The admixtures from every column and row were combined into primary pools. Derived from primary pools, superpools contained column admixtures plus row admixtures. Secondary pools which included column admixtures and row admixtures from the 40 384-well culture plates were also constructed. The recombinant pCC2FOS^TM^ vector was extracted from cell cultures combined from one set of 384-well culture plates and used for PCR screening. The superpools were constructed to determine whether the recombinants from one set of 40 384-well culture plates contained positive clones. The primary pools were used to identify which plate contained one or more positive clones while the secondary pools could determine the exact positive clones. Screening primers targeting MiSp were obtained by designing a pair of degenerate PCR primers based on two conserved polypeptides in MiSp and MaSp C-terminal domain (SRISSA and NIGQVD). After optimization of reaction conditions, a ∼200 bp fragment was obtained, from which exact sequences for primers were derived. The primers (GMiSp-CF: 5′–TTACTCAGGTGTCCTTGG–3′ and GMiSp-CR: 5′–ATTGGCTTACTGCATTCT–3′) targeted a 162 bp portion of the MiSp 3′ non-repetitive region.

**Figure 3:**
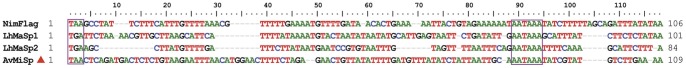
Alignment of predicted 3′ UTR sequences. **TGA/TAA** stop codons and the polyadenylation signals, AATAAA, are boxed. The UTRs are determined from genomic DNA and mRNA. Nim-UTR was determined by alignment of *N. inaurata madagascariensis* flagelliform spidroin gene (GenBank accession no. AF218623S2) and *N. clavipes* flagelliform (Flag) mRNA (GenBank accession no. AF027973); Lh-UTR 1 was determined by alignment of *L. hesperus* major ampullate spidroin 1 (MaSp1) gene (GenBank accession no. EF595246) and mRNA (GenBank accession no. DQ409057); Lh-UTR 2 was determined by alignment of *L. hesperus* major ampullate spidroin 2 (MaSp2) gene (GenBank accession no. EF595245) and mRNA (GenBank accession no. DQ409058); Av-UTR was determined by alignment of *A. ventricosus* minor ampullate spidroin gene (GenBank accession no. JX513956) and *A. ventricosus* major ampullate gland dragline silk protein-2 (F2) mRNA (GenBank accession no. AY177203).

### Full-length gene sequence

The primers pCC2FOS™ Forward Sequencing Primer: 5′–GTACAACGACACCTAGAC–3′ and the Reverse Sequencing Primer: 5′–CAGGAAACAGCCTAGGAA–3′ were designed for Fosmid end-sequencing. About 800 bp of the 5′ termini and 3′ termini of the inserted DNA were sequenced, and used to identify clones that contained full-length MiSp genes. Finally, the Chinese National Human Genome Center (CHGC) in Shanghai was commissioned to sequence the full-length gene from one positive clone, using a shotgun method. One positive clone was sequenced and assembled to ∼7×coverage. The positive plasmid was sheared randomly into 1.6–4 kb fragments and subcloned into pSMART. Subclones in two 96-well plates (192 subcloned fragments) were sequenced in two directions with ABI 3730xl DNA sequencer, yielding 384 sequenced fragments that were assembled using phredPhrap. Gaps were closed by sequencing restriction fragments. Finally the full-length sequence was verified with PCR amplification, restriction enzyme digestion and sequencing.

**Figure 4:**
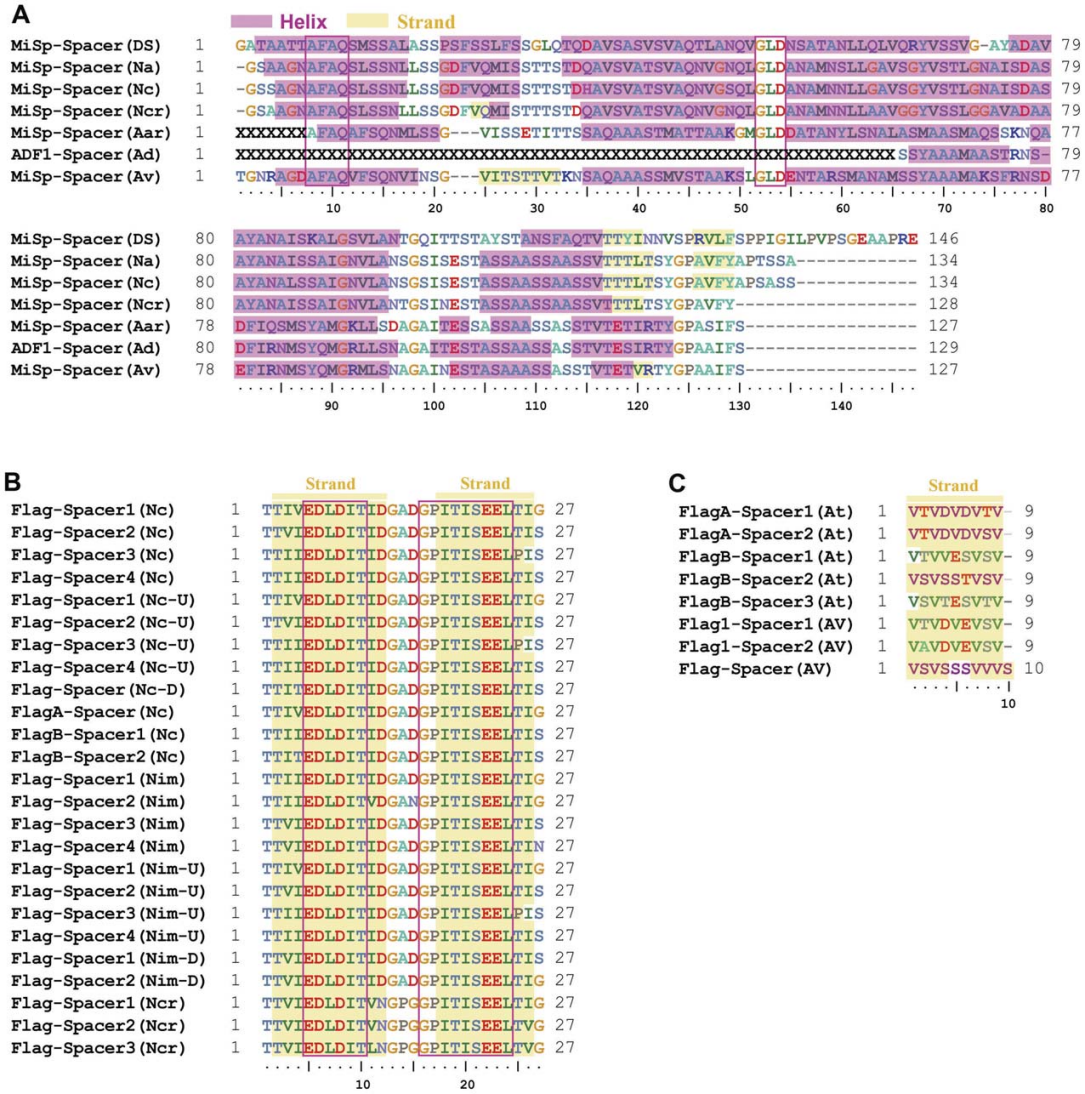
Sequence alignment of spacer regions. The spacer sequences are aligned using ClustalW2 and amino acid residues are shown in different colors. X indicates missing amino acid residues and Gaps (–) have been inserted in order to align the amino acid residues. Positions with identical residues in a majority of the proteins are boxed. α-Helices and strands predicted by PSI-Pred v3.0 are shaded in red and yellow, respectively. (A) The sequences shown are the following: MiSp-Spacer (Ds), *D. spinosa* minor ampullate spidroin (GenBank accession no. ABD61589); MiSp-Spacer (Na), *Nephila antipodiana* minor ampullate fibroin 1 (GenBank accession no. ABC72645); MiSp-Spacer (Nc), *N. clavipes* minor ampullate silk protein MiSp1 (GenBank accession no. AAC14589); MiSp-Spacer (Ncr), *Nephilengys cruentata* minor ampullate spidroin-like protein (GenBank accession no. ABR37278); MiSp-Spacer (Aar), *Argiope argentata* minor ampullate spidroin (GenBank accession no. AFM29835); ADF1-Spacer (Ad), *A. diadematus* fibroin-1 (ADF-1) (GenBank accession no. AAC47008); MiSp (Av), *A. ventricosus* minor ampullate spidroin (GenBank accession no. JX513956). (B) The *Nephila* Flag-spacer sequences shown are the following: Flag-Spacer1–4 (Nc), *N. clavipes* flagelliform silk protein (Flag) (GenBank accession no. AAF36090); Flag-Spacer1–4 (Nc-U), *N. clavipes* flagelliform silk protein (Flag) (GenBank accession no. AAF36090); Flag-Spacer (Nc-D), *N. clavipes* flagelliform silk protein (Flag), downstream (GenBank accession no. AAF36089); FlagA-Spacer (Nc), *N. clavipes* flagelliform silk protein (Flag) (GenBank accession no. AAC38846); FlagB-Spacer1–2 (Nc), *N. clavipes* flagelliform silk protein (Flag) (GenBank accession no. AAC38847); Flag-Spacer1–4 (Nim), *N. inaurata madagascariensis* flagelliform silk protein (Flag) (GenBank accession no. AAF36091); Flag-Spacer1–4 (Nim-U), *N. inaurata madagascariensis* flagelliform silk protein (Flag) (GenBank accession no. AAF36091); Flag-Spacer 1–2(Nim-D), *N. inaurata madagascariensis* flagelliform silk protein (Flag), downstream (GenBank accession no. AAF36092); Flag-Spacer1–3 (Ncr), *N. cruentata* flagelliform spidroin-like protein (GenBank accession no. ABR37273). (C) The *Argiope* and *Araneus* Flag-spacer sequences shown are the following: FlagA-Spacer1–2 (At), *A. trifasciata* flagelliform silk protein (Flag) (GenBank accession no. AF350265_1); FlagB-Spacer1–3 (At), *A. trifasciata* flagelliform silk protein (Flag) (GenBank accession no. AF350264_1); Flag1-Spacer1–2 (Av), *A. ventricosus* flagelliform silk protein-1 (Ff1) (GenBank accession no. AAT36347); Flag-Spacer (Av), *A.ventricosus* flagelliform fibroin (GenBank accession no. ABK00016).

### Intron identification

First we used Fgenesh 2.6 to predict intron cleavage sites and then reverse-PCR was used to determine the intron cleavage site. Minor ampullate glands were dissected from euthanized *A. ventricosus* individuals and flash frozen in liquid nitrogen. Total RNA was extracted according to the manufacturer's protocol by using the standard TRIzol^TM^ method. Four primers were designed based on the full-length MiSp gene sequence (Table 2). Repetitive DNA sequences are not suitable for designing primers, so the reverse primers (including the reverse-transcription primer) annealed to the nonrepetitive spacer region. Because the non-repetitive N-terminal domain is too far from the intron, sense primers annealed to the upstream repetitive region. cDNA from *A. ventricosus* was synthesized with nested-reverse transcription PCR in the following manner. Step 1, the Out-F(RT primer) was used for reverse-transcription with mRNA as template, amplifying single-stranded cDNA fragments. Step 2, with the cDNA as template, primers Out-S and Out-F(RT primer) were used to amplify double-stranded DNA fragments. Step 3, in order to improve the double-stranded DNA specificity, nested-primers, In-S and In-F were used to amplify double-stranded DNA fragments with double-stranded DNA generated in step 2 as template. Running the DNA fragment in step 2 and 3 on agarose gels showed no clear bands in step 2, but in step 3 two bands were seen. These two DNA fragments were sequenced, showing that one is amplified from the first spacer and the other one is amplified from the second spacer (see Figure 1). Sequencing reactions were performed at Beijing Genomics Institute, China.

**Figure 5:**
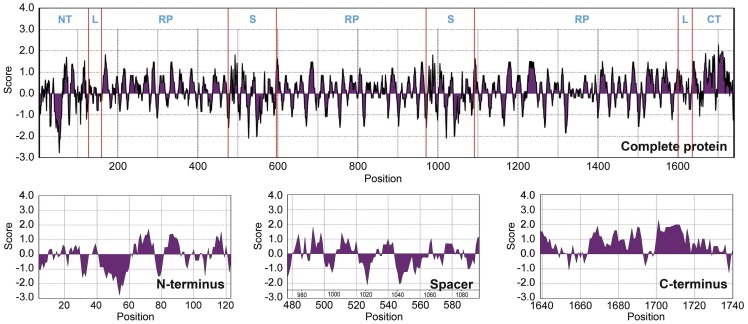
Kyte and Doolittle hydropathicity plots for *A. ventricosus* MiSp. Positive scores indicate hydrophobicity. NT = N-terminal region, RT =  repetitive region, S =  spacer and CT =  C-terminal region.

**Figure 6:**
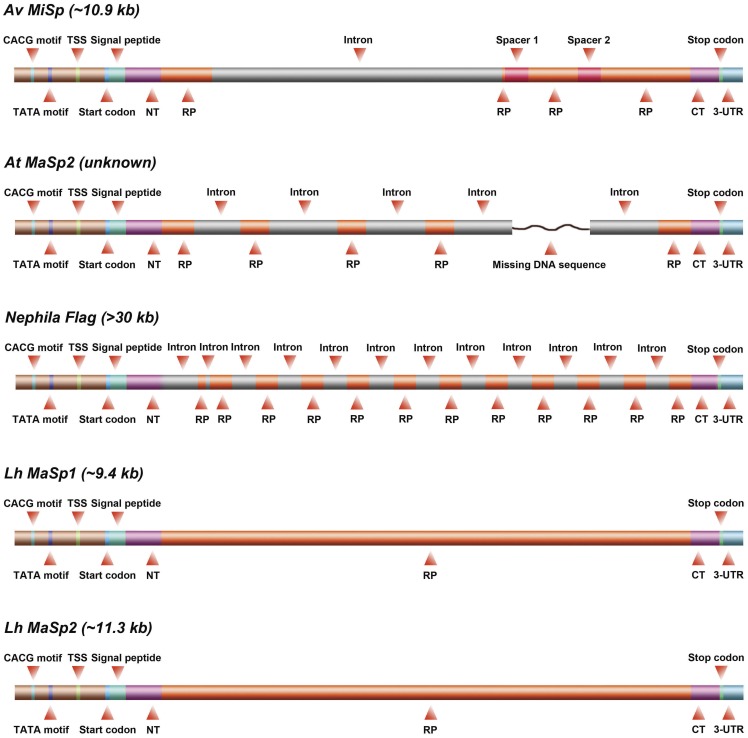
Architectures of *A. ventricosus* MiSp, *A. trifasciata* MaSp2, *Nephila* Flag, *L. hesperus* MaSp1 and MaSp2 genes. Arrangement of exons (red) interrupted by introns (grey light green) in the spidroin genes. Due to the huge variability in size the lengths of the different segments are not drawn to scale.

### Sequence analysis

Base composition, amino acid content and codon usage were analyzed using DNAman (http://www.lynnon.com/) and DNAssist [29]. Fgenesh 2.6 (http://linux1.softberry.com/berry.phtml?topic=fgenesh&group=programs&subgroup=gfind) was used for intron prediction. The Expasy tools (www.expasy.org) were used for Kyte and Doolittle hydropathicity predictions and for analysis of sequence motifs. Secondary structures were predicted by PSIPRED v3.0 (http://bioinf.cs.ucl.ac.uk/psipred/). SignalP 4.0 (http://www.cbs.dtu.dk/services/SignalP/) was used for N-terminal signal peptide prediction. Sequences were aligned by Clustalw2 (http://www.ebi.ac.uk/Tools/msa/clustalw2/) and MEGA 4 was used for phylogenetic tree construction [30].

## Results

### MiSp full-length gene and protein sequence

Two positive clones were obtained from an *A. ventricosus* Fosmid genomic library by screening with 4-dimensional PCR targeting the MiSp C-terminal region. Analysis of the 5′ and 3′ terminal ∼800 bp sequences of the inserts revealed identical sequences in both inserts and that the positive clones contained the full-length MiSp gene (data not shown). The complete ∼33 kb sequence for one of the clones, called F29-0811, was determined (GenBank accession no. JX513956) and found to encompass the full-length coding sequence for MiSp as well as 6647 bp upstream of its start codon and 14937 bp downstream of its stop codon. After removal of the single intron (see below), the full-length transcript of the MiSp gene is 5440 bp and encodes 1766 amino acid residues. The corresponding MiSp is organized into non-repetitive N- and C-terminal domains and a predominantly repetitive region in between (Figure 1).

### Species comparisons identify conserved non-coding sequences

The genomic sequences representing the 5′ end of *A.ventricosus* MiSp, *L. hesperus* MaSp1 and MaSp2, *Latrodectus geometricus* MaSp1, *Argiope trifasciata* MaSp2, *Nephila inaurata madagascariensis* MaSp2 and *N. inaurata madagascariensis* Flag genes were aligned (Figure 2). An adenine with pyrimidines in proximity is characteristic of the transcription start site (TSS) and the putative TSS (+1 position) is represented by a boxed adenine in Figure 2. The first following Met codon in frame for the *A.ventricosus* MiSp, *L.hesperus* MaSp1/2, *L. geometricus* MaSp1, *A. trifasciata* MaSp2, *N. inaurata madagascariensis* MaSp2 and *N. inaurata madagascariensis* Flag sequences occurs at position +34, +29, +29, +29 +29, +26, respectively. The approximately 500 nucleotides upstream of each Met codon are adenine- and thymine-rich and are less conserved than the coding sequence. However, some conserved motifs that likely represent regulatory elements can be discerned. In the upstream regions of the spidroin genes, there are two conserved sequence elements, TATAAAA ending between positions −24 and −26, and CACG ending between positions −43 and −51 (Figure 2). The identified TATA box motif fits the classical example in both its sequence and location with respect to predicted TSS (Figure 2), while the CACG motif does not resemble any well-characterized promoter sequence element. However, its strict conservation in all so far cloned spidroin gene sequences and its location at about −40 suggests that it might be a part of a promoter.

In the 3′untranslated regions (UTR) of *A.ventricosus* MiSp, *L.hesperus* MaSp1/2, and *N. inaurata madagascariensis* Flag, a canonical polyadenylation signal, AATAAA, is highly conserved and located at position +86, +77, +61, +77 relative to the stop codon, respectively (Figure 3).

### Identification of a large intron in the *A. ventricosus* MiSp gene

Analysis of the gene architecture using Fgenesh suggested the presence of an intron covering positions 8303 to 13930 of the genomic DNA. RT-PCR of RNA from minor ampullate glands, using reverse primers based on the spacer motif (see below) resulted in two cDNA fragments, 370 bp and 239 bp long (GenBank accession no. JX513954-55), in accordance with the presence of two identical spacer regions in MiSp (see below). The longer fragment covers upstream and downstream regions of the predicted intron, and support the existence of the Fgenesh predicted splice site. The 5′ and 3′ splice sites in the MiSp gene are located at positions 1430 and 7057, respectively. The identified intron in MiSp DNA is 5628 bp long and begins with the nucleotides of “GT” (guanine, thymine) and ends with the nucleotides “AG” (adenine, guanine) and thus follows the GT-AG rule [31], [32].

MiSp sequences from cDNA obtained by RT-PCR of mRNA from the minor ampullate glands of *A. ventricosus* reveal that the MiSp gene presented herein is transcribed and that the transcript is spliced at the predicted borders between the exons and the intron. Futhermore, the fact that we obtained two distinct cDNA sizes (370 and 239 bp) supports that there is only one MiSp gene, containing two spacers, present in *A. ventricosus.* If there are additional *A. ventricosus* MiSp genes they are either very similar in spacer sequence and –distance to the one now described, or their spacer regions are so different that the primers did not anneal to their transcripts.

### MiSp repetitive region

The translated MiSp sequence (Figure 1) shows repetitive regions composed mainly of glycine and alanine residues, which can be categorized into four types of ensemble repeat units, GX, GGX, GGGX and short poly-A repeats. The *A. ventricosus* MiSp repetitive region is distinct from previously published MiSp1 and MiSp2 repetitive sequences. Colgin et al published the first characterization of the amino acid sequence of MiSps [4] and identified two similar MiSps in *N. clavipes* (MiSp1 and MiSp2). Since then, several partial MiSps and MiSp-like proteins have been identified [8], [22], [33]–[35], but some of these sequences have not been assigned to either MiSp1 or -2 since clear and secerning classification criteria are lacking. Also the *A. ventricosus* sequence presented here is difficult to assign according to Colgin and Lewis' criteria and is therefore referred to as MiSp.

Reoccurring overall patterns can be observed in *A. ventricosus* MiSp repetitive region, but it lacks higher order organization, as seen for *L. hesperus* MaSp1 [21]. Generally, a complete repetitive unit is composed of GGXGGY-(GX)_a_(A)_b_(GX)_c_-GGAGGYGGGX-(GX)_d_ where *a = 4–13, b = 3–5, c = 0–3 and d = 1–10*. More precisely, the repetitive region of the *A. ventricosus* MiSp is dominated by the motifs: GGX, GX, poly-A (3–5 consecutive alanines) and GGGX. These motifs are organized into four types of ensemble (with order) repeat units, GGX-GGX-GX, (GX)_n_-oligoA-(GX)_n_, GGX-GGX-GGGX and (GX)_n_ (Figure 1). The repetitive region is divided into three parts by the two spacers (see below), repetitive region I, II and III. In repetitive region I the poly-A motif occurs frequently while it only occurs three times in the second repetitive region. Repetitive region III is the longest region with poly-A occuring eight times. In contrast, the poly-A motifs of MaSps are in general longer and highly repeated [21]. The poly-A motif is not even the most abundant motif in *A. ventricosus* MiSp. However, (GA)_n_ occurs frequently in MiSp while no (GA)_n_ motif is present in *L. hesperus* MaSp1 and occurs rarely in MaSp2 [21]. It is commonly believed that poly-A as well as (GA)_n_ motifs form β-sheet assemblies in the silk [26], [36], [37], so it is possible that they to some extent fulfill the same function.

The most common repeat is GX (X  = A, Q, I, V, E, S, and D) (Figure 1). The distribution of the seven residues representing X among the repeats is non-even, with GA being by far the most common variant. The second most common motif is GGX (X =  A, S, V, E, Y). The second glycine is sometimes replaced by alanine or serine and the distribution of the five X residues is non-even. The third motif, which has only been described in *Araneus diadematus* fibrion (ADF)-1 previously [38], is GGGX where X is always tyrosine or alanine. Notably, in *A. ventricosus* MiSp, repetitive motifs containing proline are absent.

Glycine (G) and alanine (A) are by far the most abundant amino acid residues in MaSp, MiSp and Flag spidroins [4], [8], [20]–[22], [33]–[35]. Glycine, alanine, serine and tyrosine are the most common amino acids in the predicted *A. ventricosus* MiSp, constituting more than 78% of the entire spidroin, and glycine and alanine constitute more than 64% (Figure S1). Because the first two codon positions for alanine and glycine are guanine or cytosine, the composition of the MiSp gene is overall guanine/cytosine-rich (58%). The third positions for glycine, alanine, serine and tyrosine codons in *A. ventricosus* MiSp is biased towards adenine and uracil (Figure S2), in accordance with the amino acid compositions and codon usage of MaSps from *L. hesperus* [21] and other silk proteins [9], [19], [39], [40].

### Two identical nonrepetitive spacer regions

The repetitive region of *A. ventricosus* MiSp is interrupted by two serine-rich spacer regions (Figure 1). The 126 residue spacer regions are identical even at the nucleotide level and show no identity to any proteins except other spacer regions in MiSp and Flag. Serine and alanine, 17% and 20% respectively, are the most abundant residues in this region, while the glycine content (6%) is much lower in the spacers than in the repetitive regions (>36%) (Figure S1). Interestingly, the spacer regions are similar in both length and amino acid composition to the N-terminal domain although they are apparently non-homologous (Figure S1). The spacer region is not internally repetitive, with the exception of the sequence, AAASS, which is present as a single tandem repeat. In the so far characterized MiSp spacers, AFAQ, GLD and SA-rich region motifs are highly conserved (Figure 4).

### Non-repetitive N- and C-terminal regions

In *A. ventricosus* MiSp, the non-repetitive N-terminal region (Figure 1) is composed of a predicted signal peptide followed by an about 130-residue domain. The latter is homologous to the five-helix bundle structure described for *Euprosthenops australis* MaSp N-terminal domain [41], as judged by sequence alignments (Figure S3). The SignalP 4.0 program predicted the presence of a signal peptide starting at Met1 and ending with a signal peptidase cleavage site [42] with a probability score of 0.807 (Figure S3). Signal peptides were not predicted following any other of the Met residues in the N-terminal region. This reinforces that the first Met represents the true translational start site of *A. ventricosus* MiSp, and that it is a secretory protein.

The *A. ventricosus* N-terminal domain sequence was analyzed by PSI-Pred v3.0 [43], which predicted five α-helices in essentially the same regions as for other N-terminal domains (Figure S3). In *A. ventricosus* MiSp N-terminal domain two cysteines are found in locations corresponding to helix 1 and 4, respectively. The locations of these Cys correspond well with those of conserved Cys in helix 1 and 4 from TuSp, CySp, MaSp2, and MiSp (Figure S3). Judging from the crystal and NMR structures of *E. australis* N-terminal domain [41], [44], the two cysteines in *A. ventricosus* MiSp N-terminal domain are localized so that they can form an intramolecular disulphide. Likewise, two cysteines present in *Deinopis spinosa* MaSp2 N-terminal domain in locations corresponding to helix 1 and 2 (Figure S3), are situated so that they can form an intramolecular disulphide.

Phylogenetic analysis based on multiple sequence alignments of N-terminal domains (Figure S3), with the predicted signal peptides removed, shows that *A. ventricosus* MiSp clusters according to glandular origin (data not shown), as previously described for other spidroins [9], [25].

As for the N-terminal domain, the *A. ventricosus* MiSp C-terminal domain is homologous to previously described C-terminal domains from other spidroins and species (Figure S4). In the MiSp C-terminal domain, alanine, glycine, serine and valine are common (about 64%), and the glycine content (about 11%) is less than a half of that in the repetitive region but about three times that in N-terminal region (Figure S1). Notably, cysteine is absent in *A. ventricosus* MiSp C-terminal domain, in contrast to most other known such domains, which form disulphide linked homodimers [39], [45]. The C-terminal domain is evolutionarily conserved, but to a lesser extent then the N-terminal domain (Figures S3 and S4). Notably, the Flag C-terminal domain is divergent, to the extent that it is difficult to align it with other such domains (Figure S4).

## Discussion

### Overall MiSp properties

The MiSp characterized herein and previously described MaSps have glycine and alanine rich motifs that occur in ensemble repeats, but the repeat organization (repetitiveness) and similarity of repeat copies (homogenization) differ. In *L. hesperus* MaSps, four types of ensemble repeats are strung together to form higher-level repeat units, that can be tandemly arrayed up to twenty times and the iterations share high identity at both the amino acid and nucleotide level [21]. *A.ventricosus* MiSp shows more sequence and length variation among its ensemble repeats than *L.hesperus* MaSps. The modular architectures of *A.ventricosus* MiSp and *L.hesperus* MaSps likely reflect concerted evolution within a single gene, as has been implicated in maintaining similarity among Flag (440 residues) ensemble repeats and the long repeats of tubuliform spidrion (TuSp1, 200 residues), aciniform spidrion (AcSp1, 200 residues) and pyriform spidroin (PySp, >200 residues) [9], [11], [15], [16], [40], [46].

Hydropathy profiles predicted according to the method of Kyte and Doolittle [47] show an alternating profile for essentially the entire *A. ventricosus* MiSp, where the regions of hydrophobicity correspond to the poly-A motifs and hydrophilic regions correspond to the glycine-rich regions (Figure 5). A closer inspection, however, indicates that the spacers are mainly hydrophilic while the C-terminal domain is overall mainly hydrophobic. The latter likely reflects the ability of the C-terminal domain to dimerize through hydrophobic interactions [48]. The N-terminal domain shows pronounced hydrophilicity in the region between residues 40 and 60, in agreement with its ability to promote spidroin solubility at neutral pH [41].

### Variable spidroin gene architectures

The two MiSp spacer regions are identical even at the nucleotide level, a phenomenon previously not observed for any spidroin gene. Moreover, in contrast to the long MiSp spacers the spacers of *Nephila* and *Nephilengys* Flag are only 27 residues (Figure 4B) and the spacers of *Araneus* and *Argiope* Flag are even shorter, just 9 residues (Figure 4C). *Nephila* and *Nephilengys* Flag spacers contain highly conserved motifs EDLDIT and GPITISEEL while the short *Araneus* and *Argiope* Flag spacers are very rich in valine, but lack longer conserved motifs (Figure 4B and 4C). Not only does the amino acid sequences differ between MiSp and Flag spacer regions, their predicted secondary structures are entirely different. While the MiSp spacers are predicted to contain mainly long stretches of α-helical structure, both the 27- and 9-residue Flag spacers are predicted to contain β-strands only (Figure 4).

The *A.ventricosus* MiSp gene is the first characterized spider silk gene with a single intron (Figure 6). In contrast, the previously described MiSp cDNA fragments and genomic DNA restriction mapping of *N. clavipes* show no detectable introns [4]. In line with this, *L. hesperus* MaSp1/2 full-length genomic DNA sequences, and *L. geometricus* MaSp1 and *Nephila* MaSp2 fragments reveal that these spider silk genes are composed of a single large exon [21], [24]. Also the TuSp1, AcSp and PySp genes are suggested to contain single exons [40], [49]. On the other hand, the *A. trifasciata* MaSp2 gene and the Flag gene contain multiple introns that are almost identical within the respective gene (Figure S5) [20], [24]. Thus, all known spider silk genes have different exon-intron architectures, also within a specific spider silk gene (*eg* MaSp2). Once an intron invades a gene, the intron can be rapidly propagated throughout the gene, a phenomenon that appears to be common in silk genes [20], [40], [50]. However, for the *A.ventricosus* MiSp gene this is not the case. In general, the introns within a spider silk gene show high degree of similarity, but between different silk genes the introns are more diverse (Figure S5). In line with this, the *A.ventricosus* MiSp intron differs from the Flag and MaSp2 introns both in terms of size and sequence (Figure 6 and Figure S5).

Many eukaryotic genes are intronless [51], but proteins encoded by single exons are strongly biased towards smaller sizes (less than 1000 amino acids) [52], [53]. Spider silk genes are not typical in this sense, *eg* the intronless *L. hesperus MaSp1/2* genes code for 3129 and 3779 amino acid residues, respectively [21], while the intron-containing gene for *A.ventricosus* MiSp codes for 1766 amino acids. Also, the MiSp intron is large, 5628 bp, compared to other silk genes (496–2620 bp, Figure 6 and Figure S5). Generally, intron length is negatively correlated with expression level [54]–[56], but minor ampullate silk genes are likely highly expressed.

In conclusion, determination of the first full-length MiSp gene sequence reveals an unusually variable repetitive part, extremely conserved spacer regions, and that the exon/intron organization differs between all so far characterized spidroin genes.

## Supporting Information

Figure S1 **Amino acid composition of different regions of** ***A. ventricosus*** **MiSp.** Amino acid composition of complete *A. ventricosus* MiSp (A), its N-terminal domain (B), its C-terminal domain (C), and its spacer sequences (D).
(TIF)

Figure S2 **Codon usage for the most common amino acids of** ***A. ventricosus*** **MiSp.**
(TIF)

Figure S3 **Sequence alignment of all reported nonrepetitive N-terminal regions.** The nonrepetitive N-terminal sequences are aligned by ClustalW2 and amino acids are shown in different colors. Signal peptides predicted with SignalP 4.0 are shaded in blue. α-helices predicted with PSI-Pred v3.0 are shaded in red. The red triangle indicates *A. ventricosus* MiSp N-terminal sequence. The sequences shown are the following: TuSp1 (Aar), *A. argentata* tubuliform spidroin 1, residues 1–132 (GenBank accession no. ADM14332); TuSp1 (Na), *N. antipodiana* eggcase silk protein, residues 1–159 (GenBank accession no. ACI23395); TuSp1 (Aap), *Agelenopsis aperta* tubuliform spidroin 1, residues 1–161 (GenBank accession no. ADM14330); TuSp1 (Lh), *L. hesperus* tubuliform spidroin 1, residues 1–156 (GenBank accession no. ABD24296); CySp1 (Ab), *A. bruennichi* cylindriform spidroin 1, residues 1–154 (GenBank accession no. GenBank: BAE86855); CySp2 (Ab), *A. bruennichi* egg case silk protein 2, residues 1–96 (GenBank accession no. BAE86856, modified as in[^25^]); CySp1 (Ncl), *Nephila clavata* cylindriform spidroin 1, residues 1–159 (GenBank accession no. BAE54451). Flag (Nc), *N. clavipes* flagelliform silk protein, residues 2–153 (GenBank accession no. GenBank: AAC38846, modified as in[^25^]). Flag (Nim), *N. inaurata madagascariensis* flagelliform silk protein, residues 1–154 (GenBank accession no. GenBank: AAF36091, modified as in[^25^]). MaSp1 (Lh), *L. hesperus* major ampullate spidroin 1, residues 1–151 (GenBank accession no. ABR68856); MaSp2 (Lh), *L. hesperus* major ampullate spidroin 2, residues 1–151 (GenBank accession no. ABR68855); MaSp2 (Lg), *L. geometricus* major ampullate spidroin 2, residues 1–124 (GenBank accession no. ABY67417); MaSp1-like (Lg), *L. geometricus* major ampullate spidroin 1-like, residues 1–151 (GenBank accession no. AAZ15320, modified as in[^25^]); MaSp1 (Lg), *L. geometricus* major ampullate spidroin 1 variant 1 locus 2, residues 1–151 (GenBank accession no. ABY67428); MaSp1 (Lm), *Latrodectus mactans* major ampullate spidroin 1-like, residues 1–127 (GenBank accession no. ADO78764); MaSp1B (Nc), *N. clavipes* major ampullate spidroin 1B precursor, residues 1–154 (GenBank accession no. ACF19412); MaSp1A (Nc), *N. clavipes* major ampullate spidroin 1A precursor, residues 1–154 (GenBank accession no. ACF19411); MaSp2 (Nc), *N. clavipes* major ampullate spidroin 2 precursor, residues 1–154 (GenBank accession no. ACF19413); MaSp2-like (Nim), *N. inaurata madagascariensis* major ampullate spidroin 2-like, residues 1–154 (GenBank accession no. AAZ15322); MaSp2 (At), *A. trifasciata* major ampullate spidroin 2, residues 1–155 (GenBank accession no. AAZ15371); MaSp (Aap), *A. aperta* major ampullate spidroin, residues 1–151 (GenBank accession no. ADM14324); MaSp2 (Ds), *D. spinosa* major ampullate spidroin 2, residues 3–148 (GenBank accession no. ADM14319); MaSp(Dc), *Diguetia canities* major ampullate spidroin, residues 1–161 (GenBank accession no. ADM14315); MaSp-like(Dc), *D. canities* major ampullate spidroin-like protein, residues 1–147 (GenBank accession no. ADM14317.1); MaSp1(Ea), *E. australis* major ampullate spidroin 1 precursor, residues 1–154 (GenBank accession no. CAJ90517); MaSp1(Kh), *Kukulcania hibernalis* major ampullate spidroin 1, residues 10–156 (GenBank accession no. ADM14314); MiSp(Mg), *Metepeira grandiosa* minor ampullate spidroin, residues 1–143 (GenBank accession no. ADM14328); MiSp(Ud), *Uloborus diversus* minor ampullate spidroin, residues 1–141 (GenBank accession no. ADM14326); MiSp(Lh), *L. hesperus* minor ampullate spidroin, residues 1–151 (GenBank accession no. ADM14321); MiSp (Av), *A. ventricosus* minor ampullate spidroin, residues 1–149 (GenBank accession no. JX513956).
(TIF)

Figure S4 **Sequence alignment of nonrepetitive C-terminal regions.** The nonrepetitive C-terminal sequences are aligned by ClustalW2 using default parameters and amino acids are shown in different colors. α-helices and strands predicted with PSI-Pred v3.0 are shaded in red and yellow, respectively. The red triangle indicates *A. ventricosus* MiSp C-terminal sequence. Cysteines are in box. The sequences shown are the following: MaSp(Dc), *D. canities* major ampullate spidroin (GenBank accession no. ADM14316); MaSp-like(Dc), *D. canities* major ampullate spidroin-like protein (GenBank accession no. ADM14318); MaSp2A (Ds), *D. spinosa* major ampullate spidroin 2a (GenBank accession no. ABD61593); MaSp2B (Ds), *D. spinosa* major ampullate spidroin 2b (GenBank accession no. ABD61594); MaSp (Aap), *A. aperta* major ampullate spidroin (GenBank accession no. AAT08436); MaSp1(Ea), *E. australis* major ampullate spidroin 1 (GenBank accession no. CAJ00428); MaSp2 (Nc), *N. clavipes* major ampullate spidroin 2 (GenBank accession no. AAT75317); MaSp2 (Nim), *N. inaurata madagascariensis* major ampullate spidroin 2 (GenBank accession no. AF350278_1); MaSp1A (Nc), *N. clavipes* major ampullate spidroin 1 (GenBank accession no. AAT75312); MaSp1B (Nc), *N. clavipes* major ampullate spidroin 1 (GenBank accession no. AAT75311); MaSp2 (At), *A. trifasciata* major ampullate spidroin 2 (GenBank accession no. AAZ15372); MaSp1 (Lh), *L. hesperus* major ampullate spidroin 1 (GenBank accession no. ABR68856); MaSp1 (Lg), *L. geometricus* major ampullate spidroin 1 (GenBank accession no. AF350273_1); MaSp1-like (Lg), *L. geometricus* major ampullate spidroin 1-like (GenBank accession no. AAZ15321); MaSp2 (Lh), *L. hesperus* major ampullate spidroin 2 (GenBank accession no. ABR68855); MaSp2 (Lg), *L. geometricus* major ampullate spidroin 2 (GenBank accession no. AF350275_1); MiSp(Ds), *D. spinosa* minor ampullate spidroin (GenBank accession no. ABD61589); MiSp(Lh), *L. hesperus* minor ampullate spidroin (GenBank accession no. ADM14322); MiSp-like(Lh), *L. hesperus* minor ampullate spidroin 1-like protein (GenBank accession no. ACB29694); MiSp1(Nc), *N. clavipes* minor ampullate silk protein 1 (GenBank accession no. AAC14589); MiSp1(Na), *N. antipodiana* minor ampullate fibroin 1 (GenBank accession no. ABC72645); MiSp(Nc), *N. clavipes* minor ampullate silk protein (GenBank accession no. AAC14590); MiSp(Ud), *U. diversus* minor ampullate spidroin (GenBank accession no. ABD61597); MiSp (Av), *A. ventricosus* minor ampullate spidroin (GenBank accession no. JX513956); ADF1(Ad), *A. diadematus* fibroin-1 (GenBank accession no. AAC47008); MiSp(Mg), *M. grandiosa* minor ampullate spidroin (GenBank accession no. ADM14320); MiSp(Aar), *A. argentata* minor ampullate spidroin (GenBank accession no. AFM29836); MiSp-like(Pb), *Parawixia bistriata* MiSp-like protein (GenBank accession no. ADG57595); TuSp1 (Aap), *A. aperta* tubuliform spidroin 1 (GenBank accession no. ADM14323); TuSp1 (Lh), *L. hesperus* tubuliform spidroin 1 (GenBank accession no. AAY28931); TuSp1 (Aar), *A. argentata* tubuliform spidroin 1 (GenBank accession no. AAY28932); CySp1 (Ab), *A. bruennichi* cylindriform spidroin 1 (GenBank accession no. BAE86855); CySp1 (Ncl), *N. clavata* cylindrical silk protein 1 (GenBank accession no. BAE54450); Flag (Nc), *N. clavipes* flagelliform silk protein (GenBank accession no. AAC38847). Flag (Nim), *N. inaurata madagascariensis* flagelliform silk protein (GenBank accession no. AAF36092).
(TIF)

Figure S5 **Percent pairwise sequence identities between introns from** ***A. ventricosus*** **MiSp,** ***A. trifasciata*** **MaSp2, and** ***N. clavipes*** **and** ***N. inaurata madagascariensis*** **Flag spider genes.** The scores within the same gene sequences are shaded in blue. Scores over 80 are bold. The red triangle indicates *A. ventricosus* MiSp intron. The sequences shown are the following: MiSp-intron (Av) is from *A. ventricosus* minor ampullate spidroin gene sequence (GenBank accession no. JX513956); MaSp2-intron1∼4 (At) is from *A. trifasciata* major ampullate spidroin 2 gene (GenBank accession no. DQ059136S1); MaSp2-intronIc (At) is from *A. trifasciata* major ampullate spidroin 2 gene (GenBank accession no. DQ059136S2); Flag-intron1∼6(Nc-U) is from *N. clavipes* flagelliform silk protein (Flag) gene (GenBank accession no. AF218621S1); Flag-intron1 (Nc-D) is from *N. clavipes* flagelliform silk protein (Flag) gene (GenBank accession no. AF218621S2); Flag-intron1∼6(Nim-U) is from *N. inaurata madagascariensis* flagelliform silk protein (Flag) gene (GenBank accession no. AF218623S1); Flag-intron (Nim-D) is from *N. inaurata madagascariensis* flagelliform silk protein (Flag) gene (GenBank accession no. AF218623S2).
(TIF)

## References

[1] Foelix RF (1996) Biology of Spiders. New York: Oxford University Press.

[2] Gosline JM, Guerette PA, Ortlepp CS, Savage KN (1999) The mechanical design of spider silks: from fibroin sequence to mechanical function. J Exp Biol 202: 3295–3303. 10562512 10.1242/jeb.202.23.3295

[3] Gosline JM, Demont ME, Denny MW (1986) The Structure and Properties of Spider Silk. Endeavour 10: 37–43.

[4] Colgin MA, Lewis RV (1998) Spider minor ampullate silk proteins contain new repetitive sequences and highly conserved non-silk-like “spacer regions”. Protein Sci 7: 667–672. 9541398 10.1002/pro.5560070315 PMC2143960

[5] Stauffer SL, Coguill SL, Lewis RV (1994) Comparison of Physical Properties of Three Silks from Nephila clavipes and Araneus gemmoides. The Journal of Arachnology 22: 5–11.

[6] La Mattina C, Reza R, Hu X, Falick AM, Vasanthavada K, et al (2008) Spider minor ampullate silk proteins are constituents of prey wrapping silk in the cob weaver Latrodectus hesperus. Biochemistry 47: 4692–4700. 18376847 10.1021/bi800140q

[7] Work RW (1977) Dimensions, Birefringences, and Force-Elongation Behavior of Major and Minor Ampullate Silk Fibers from Orb-Web-Spinning Spiders – The Effects of Wetting on these Properties. Textile Research Journal 47: 650–662.

[8] Guinea GV, Elices M, Plaza GR, Perea GB, Daza R, et al (2012) Minor Ampullate Silks from Nephila and Argiope Spiders: Tensile Properties and Microstructural Characterization. Biomacromolecules 13: 2087–2098. 22668322 10.1021/bm3004644

[9] Hayashi CY, Lewis RV (1998) Evidence from flagelliform silk cDNA for the structural basis of elasticity and modular nature of spider silks. Journal of Molecular Biology 275: 773–784. 9480768 10.1006/jmbi.1997.1478

[10] Gosline J, Lillie M, Carrington E, Guerette P, Ortlepp C, et al (2002) Elastic proteins: biological roles and mechanical properties. Philos Trans R Soc Lond B Biol Sci 357: 121–132. 11911769 10.1098/rstb.2001.1022 PMC1692928

[11] Hayashi CY, Blackledge TA, Lewis RV (2004) Molecular and mechanical characterization of aciniform silk: uniformity of iterated sequence modules in a novel member of the spider silk fibroin gene family. Mol Biol Evol 21: 1950–1959. 15240839 10.1093/molbev/msh204

[12] Gellynck K, Verdonk P, Forsyth R, Almqvist KF, Van Nimmen E, et al (2008) Biocompatibility and biodegradability of spider egg sac silk. J Mater Sci Mater Med 19: 2963–2970. 18360800 10.1007/s10856-007-3330-0

[13] Tian M, Lewis RV (2005) Molecular characterization and evolutionary study of spider tubuliform (eggcase) silk protein. Biochemistry 44: 8006–8012. 15924419 10.1021/bi050366u

[14] Viney C (2000) From natural silks to new polymer fibres. Journal of the Textile Institute 91: 2–23.

[15] Perry DJ, Bittencourt D, Siltberg-Liberles J, Rech EL, Lewis RV (2010) Piriform Spider Silk Sequences Reveal Unique Repetitive Elements. Biomacromolecules 11: 3000–3006. 20954740 10.1021/bm1007585 PMC3037428

[16] Blasingame E, Tuton-Blasingame T, Larkin L, Falick AM, Zhao L, et al (2009) Pyriform Spidroin 1, a Novel Member of the Silk Gene Family That Anchors Dragline Silk Fibers in Attachment Discs of the Black Widow Spider, Latrodectus hesperus. Journal of Biological Chemistry 284: 29097–29108. 19666476 10.1074/jbc.M109.021378 PMC2781455

[17] Vollrath F, Fairbrother WJ, Williams RJP, Tillinghast EK, Bernstein DT, et al (1990) Compounds in the Droplets of the Orb Spiders Viscid Spiral. Nature 345: 526–528.

[18] Choresh O, Bayarmagnai B, Lewis RV (2009) Spider web glue: two proteins expressed from opposite strands of the same DNA sequence. Biomacromolecules 10: 2852–2856. 19731928 10.1021/bm900681w

[19] Xu M, Lewis RV (1990) Structure of a protein superfiber: spider dragline silk. Proc Natl Acad Sci U S A 87: 7120–7124. 2402494 10.1073/pnas.87.18.7120 PMC54695

[20] Hayashi CY, Lewis RV (2000) Molecular architecture and evolution of a modular spider silk protein gene. SCIENCE 287: 1477–1479. 10688794 10.1126/science.287.5457.1477

[21] Ayoub NA, Garb JE, Tinghitella RM, Collin MA, Hayashi CY (2007) Blueprint for a high-performance biomaterial: full-length spider dragline silk genes. PLoS ONE 2: e514. 17565367 10.1371/journal.pone.0000514 PMC1885213

[22] Gatesy J, Hayashi C, Motriuk D, Woods J, Lewis R (2001) Extreme diversity, conservation, and convergence of spider silk fibroin sequences. SCIENCE 291: 2603–2605. 11283372 10.1126/science.1057561

[23] Pouchkina-Stantcheva NN, McQueen-Mason SJ (2004) Molecular studies of a novel dragline silk from a nursery web spider, Euprosthenops sp. (Pisauridae). Comp Biochem Physiol B Biochem Mol Biol 138: 371–376. 15325337 10.1016/j.cbpc.2004.04.020

[24] Motriuk-Smith D, Smith A, Hayashi CY, Lewis RV (2005) Analysis of the conserved N-terminal domains in major ampullate spider silk proteins. Biomacromolecules 6: 3152–3159. 16283740 10.1021/bm050472b

[25] Rising A, Hjalm G, Engstrom W, Johansson J (2006) N-terminal nonrepetitive domain common to dragline, flagelliform, and cylindriform spider silk proteins. Biomacromolecules 7: 3120–3124. 17096540 10.1021/bm060693x

[26] Hayashi CY, Shipley NH, Lewis RV (1999) Hypotheses that correlate the sequence, structure, and mechanical properties of spider silk proteins. Int J Biol Macromol 24: 271–275. 10342774 10.1016/s0141-8130(98)00089-0

[27] Zhao AC, Zhao TF, Nakagaki K, Zhang YS, Sima YH, et al (2006) Novel molecular and mechanical properties of egg case silk from wasp spider, Argiope bruennichi. Biochemistry 45: 3348–3356. 16519529 10.1021/bi052414g

[28] Asakawa S, Abe I, Kudoh Y, Kishi N, Wang Y, et al (1997) Human BAC library: construction and rapid screening. Gene 191: 69–79. 9210591 10.1016/s0378-1119(97)00044-9

[29] Patterton HG, Graves S (2000) DNAssist: the integrated editing and analysis of molecular biology sequences in windows. Bioinformatics 16: 652–653. 11038336 10.1093/bioinformatics/16.7.652

[30] Tamura K, Dudley J, Nei M, Kumar S (2007) MEGA4: Molecular Evolutionary Genetics Analysis (MEGA) software version 4.0. Molecular Biology and Evolution 24: 1596–1599. 17488738 10.1093/molbev/msm092

[31] Stephens RM, Schneider TD (1992) Features of spliceosome evolution and function inferred from an analysis of the information at human splice sites. J Mol Biol 228: 1124–1136. 1474582 10.1016/0022-2836(92)90320-j

[32] Breathnach R, Benoist C, O'Hare K, Gannon F, Chambon P (1978) Ovalbumin gene: evidence for a leader sequence in mRNA and DNA sequences at the exon-intron boundaries. Proc Natl Acad Sci U S A 75: 4853–4857. 283395 10.1073/pnas.75.10.4853 PMC336219

[33] Bittencourt D, Souto BM, Verza NC, Vinecky F, Dittmar K, et al (2007) Spidroins from the Brazilian spider Nephilengys cruentata (Araneae: Nephilidae). Comp Biochem Physiol B Biochem Mol Biol 147: 597–606. 17490908 10.1016/j.cbpb.2007.03.013

[34] Garb JE, Ayoub NA, Hayashi CY (2010) Untangling spider silk evolution with spidroin terminal domains. BMC Evol Biol 10: 243. 20696068 10.1186/1471-2148-10-243 PMC2928236

[35] Garb JE, Dimauro T, Vo V, Hayashi CY (2006) Silk genes support the single origin of orb webs. SCIENCE 312: 1762. 16794073 10.1126/science.1127946

[36] Parkhe AD, Seeley SK, Gardner K, Thompson L, Lewis RV (1997) Structural studies of spider silk proteins in the fiber. J Mol Recognit 10: 1–6. 9179774 10.1002/(SICI)1099-1352(199701/02)10:1<1::AID-JMR338>3.0.CO;2-7

[37] Bram A, Branden CI, Craig C, Snigireva I, Riekel C (1997) X-ray diffraction from single fibres of spider silk. Journal of Applied Crystallography 30: 390–392.

[38] Guerette PA, Ginzinger DG, Weber BH, Gosline JM (1996) Silk properties determined by gland-specific expression of a spider fibroin gene family. SCIENCE 272: 112–115. 8600519 10.1126/science.272.5258.112

[39] Rising A, Johansson J, Larson G, Bongcam-Rudloff E, Engstrom W, et al (2007) Major ampullate spidroins from Euprosthenops australis: multiplicity at protein, mRNA and gene levels. Insect Mol Biol 16: 551–561. 17680798 10.1111/j.1365-2583.2007.00749.x

[40] Garb JE, Hayashi CY (2005) Modular evolution of egg case silk genes across orb-weaving spider superfamilies. Proceedings of the National Academy of Sciences of the United States of America 102: 11379–11384. 16061817 10.1073/pnas.0502473102 PMC1183556

[41] Askarieh G, Hedhammar M, Nordling K, Saenz A, Casals C, et al (2010) Self-assembly of spider silk proteins is controlled by a pH-sensitive relay. Nature 465: 236–238. 20463740 10.1038/nature08962

[42] Petersen TN, Brunak S, von Heijne G, Nielsen H (2011) SignalP 4.0: discriminating signal peptides from transmembrane regions. Nat Methods 8: 785–786. 21959131 10.1038/nmeth.1701

[43] Jones DT (1999) Protein secondary structure prediction based on position-specific scoring matrices. Journal of Molecular Biology 292: 195–202. 10493868 10.1006/jmbi.1999.3091

[44] Jaudzems K, Askarieh G, Landreh M, Nordling K, Hedhammar M, et al (2012) pH-Dependent Dimerization of Spider Silk N-Terminal Domain Requires Relocation of a Wedged Tryptophan Side Chain. J Mol Biol. 422: 477–487. 10.1016/j.jmb.2012.06.004 22706024

[45] Hagn F, Eisoldt L, Hardy JG, Vendrely C, Coles M, et al (2010) A conserved spider silk domain acts as a molecular switch that controls fibre assembly. Nature 465: 239–242. 20463741 10.1038/nature08936

[46] Geurts P, Zhao L, Hsia Y, Gnesa E, Tang S, et al (2010) Synthetic spider silk fibers spun from Pyriform Spidroin 2, a glue silk protein discovered in orb-weaving spider attachment discs. Biomacromolecules 11: 3495–3503. 21053953 10.1021/bm101002w

[47] Kyte J, Doolittle RF (1982) A simple method for displaying the hydropathic character of a protein. J Mol Biol 157: 105–132. 7108955 10.1016/0022-2836(82)90515-0

[48] Sponner A, Vater W, Rommerskirch W, Vollrath F, Unger E, et al (2005) The conserved C-termini contribute to the properties of spider silk fibroins. Biochem Biophys Res Commun 338: 897–902. 16253207 10.1016/j.bbrc.2005.10.048

[49] Lin Z, Huang W, Zhang J, Fan JS, Yang D (2009) Solution structure of eggcase silk protein and its implications for silk fiber formation. Proc Natl Acad Sci U S A 106: 8906–8911. 19458259 10.1073/pnas.0813255106 PMC2690042

[50] Beckwitt R, Arcidiacono S, Stote R (1998) Evolution of repetitive proteins: spider silks from Nephila clavipes (Tetragnathidae) and Araneus bicentenarius (Araneidae). Insect Biochem Mol Biol 28: 121–130. 9654736 10.1016/s0965-1748(97)00083-0

[51] Gilbert W (1978) Why genes in pieces? Nature 271: 501. 622185 10.1038/271501a0

[52] Sakharkar MK, Kangueane P, Petrov DA, Kolaskar AS, Subbiah S (2002) SEGE: A database on ‘intron less/single exonic’ genes from eukaryotes. Bioinformatics 18: 1266–1267. 12217920 10.1093/bioinformatics/18.9.1266

[53] Sakharkar MK, Kangueane P (2004) Genome SEGE: A database for ‘intronless’ genes in eukaryotic genomes. Bmc Bioinformatics 5: 1–5. 15175116 10.1186/1471-2105-5-67 PMC434494

[54] Castillo-Davis CI, Mekhedov SL, Hartl DL, Koonin EV, Kondrashov FA (2002) Selection for short introns in highly expressed genes. Nat Genet 31: 415–418. 12134150 10.1038/ng940

[55] Urrutia AO, Hurst LD (2003) The signature of selection mediated by expression on human genes. Genome Res 13: 2260–2264. 12975314 10.1101/gr.641103 PMC403694

[56] Marais G, Nouvellet P, Keightley PD, Charlesworth B (2005) Intron size and exon evolution in Drosophila. Genetics 170: 481–485. 15781704 10.1534/genetics.104.037333 PMC1449718

