# Supplementary figures and images for: Full-Length Minor Ampullate Spidroin Gene Sequence
Source: PLoS One. 2012 Dec 14;7(12):e52293. doi: 10.1371/journal.pone.0052293 (PMC3522626; doi:10.1371/journal.pone.0052293)

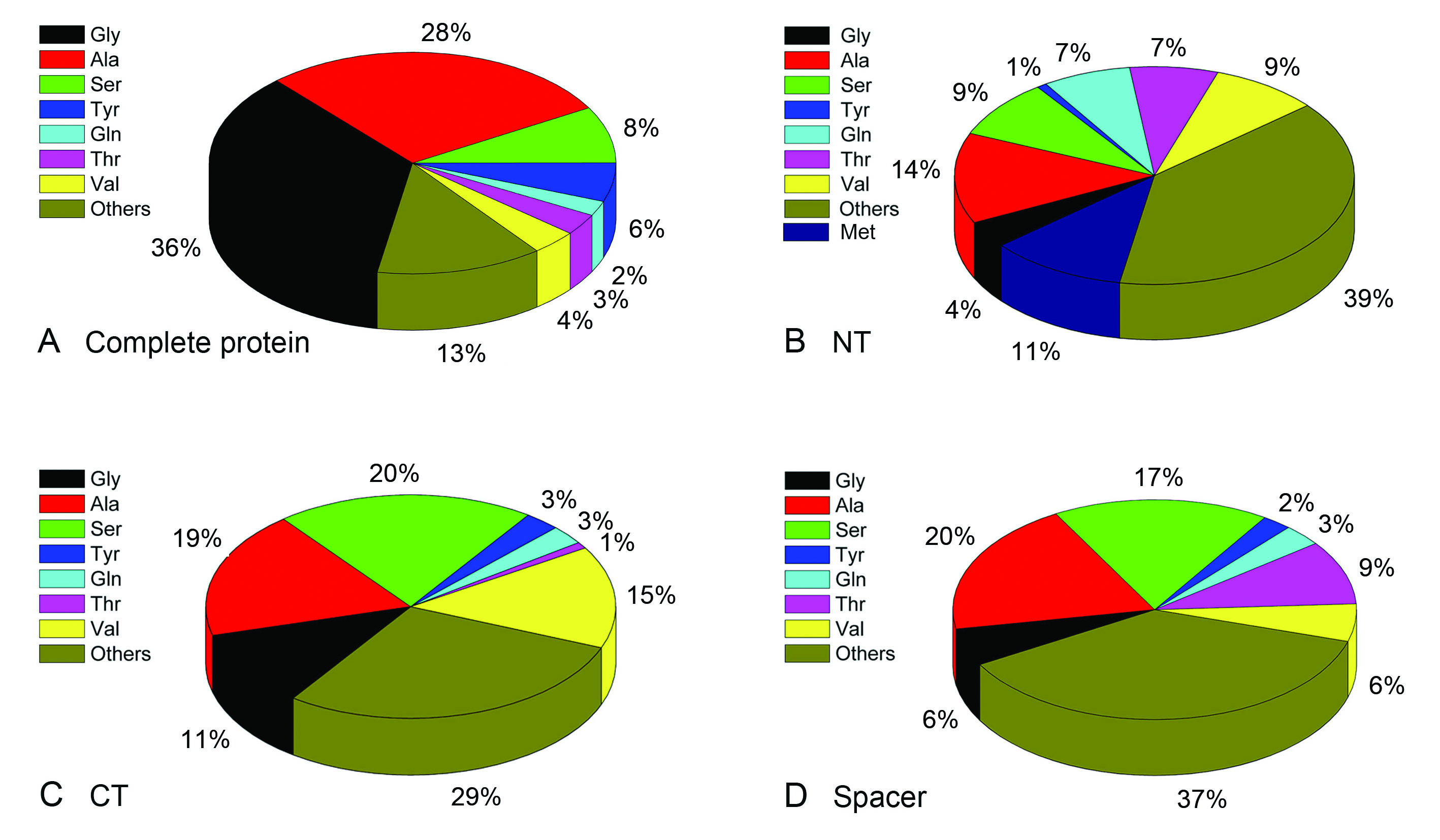

Supplement: Figure S1 — Amino acid composition of different regions of A. ventricosus MiSp. Amino acid composition of complete A. ventricosus MiSp (A), its N-terminal domain (B), its C-terminal domain (C), and its spacer sequences (D). (TIF) [file pone.0052293.s001.tif]

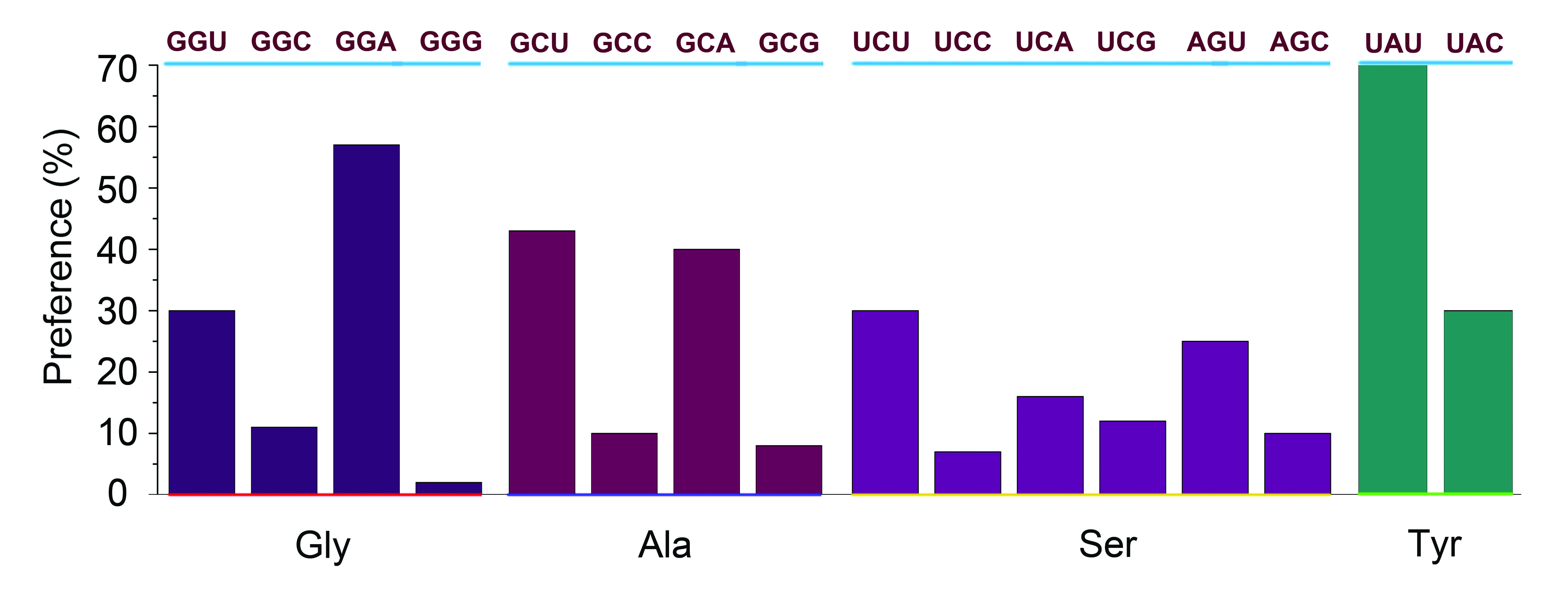

Supplement: Figure S2 — Codon usage for the most common amino acids of A. ventricosus MiSp. (TIF) [file pone.0052293.s002.tif]

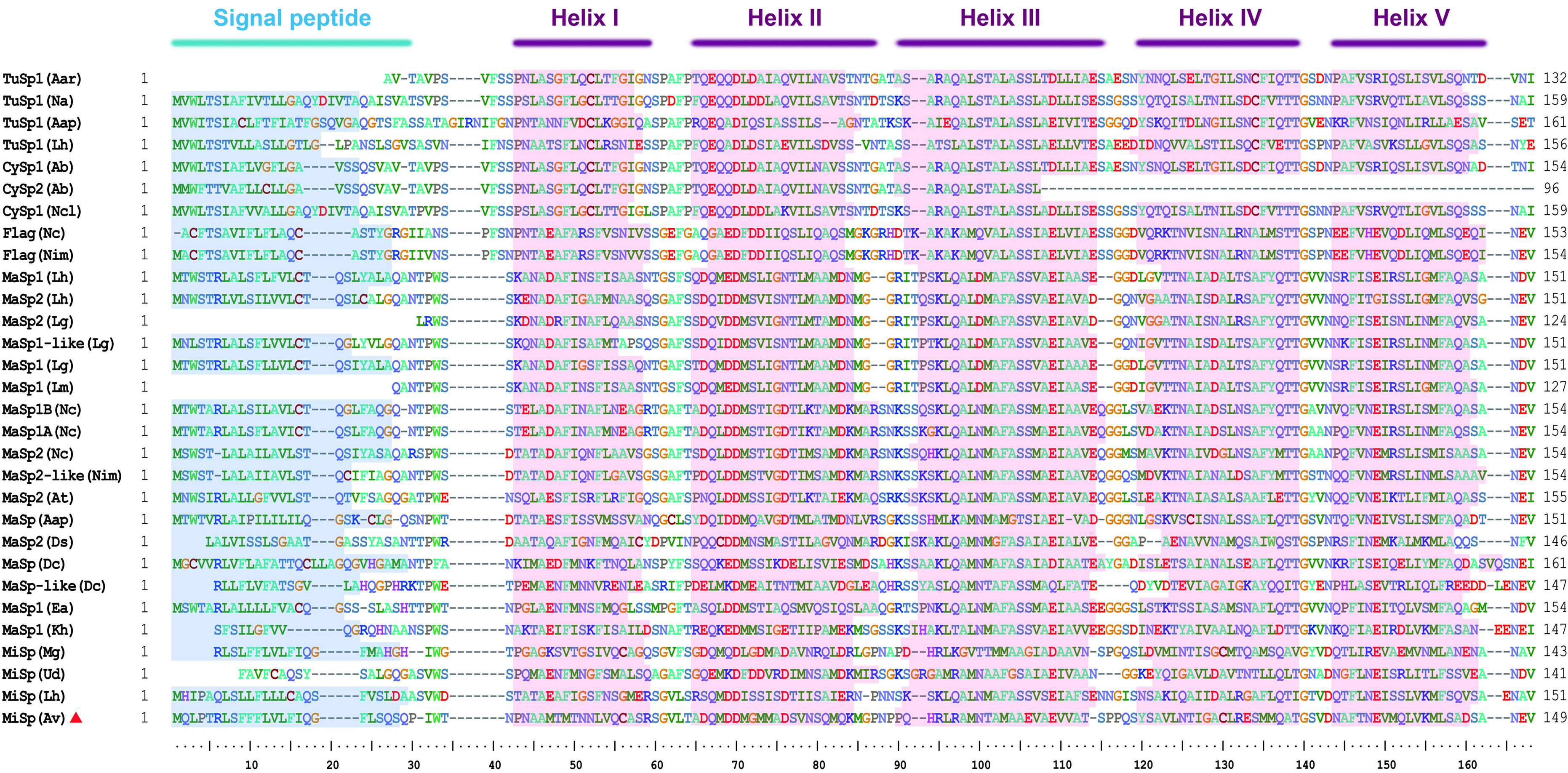

Supplement: Figure S3 — Sequence alignment of all reported nonrepetitive N-terminal regions. The nonrepetitive N-terminal sequences are aligned by ClustalW2 and amino acids are shown in different colors. Signal peptides predicted with SignalP 4.0 are shaded in blue. α-helices predicted with PSI-Pred v3.0 are shaded in red. The red triangle indicates A. ventricosus MiSp N-terminal sequence. The sequences shown are the following: TuSp1 (Aar), A. argentata tubuliform spidroin 1, residues 1–132 (GenBank accession no. ADM14332); TuSp1 (Na), N. antipodiana eggcase silk protein, residues 1–159 (GenBank accession no. ACI23395); TuSp1 (Aap), Agelenopsis aperta tubuliform spidroin 1, residues 1–161 (GenBank accession no. ADM14330); TuSp1 (Lh), L. hesperus tubuliform spidroin 1, residues 1–156 (GenBank accession no. ABD24296); CySp1 (Ab), A. bruennichi cylindriform spidroin 1, residues 1–154 (GenBank accession no. GenBank: BAE86855); CySp2 (Ab), A. bruennichi egg case silk protein 2, residues 1–96 (GenBank accession no. BAE86856, modified as in[25]); CySp1 (Ncl), Nephila clavata cylindriform spidroin 1, residues 1–159 (GenBank accession no. BAE54451). Flag (Nc), N. clavipes flagelliform silk protein, residues 2–153 (GenBank accession no. GenBank: AAC38846, modified as in[25]). Flag (Nim), N. inaurata madagascariensis flagelliform silk protein, residues 1–154 (GenBank accession no. GenBank: AAF36091, modified as in[25]). MaSp1 (Lh), L. hesperus major ampullate spidroin 1, residues 1–151 (GenBank accession no. ABR68856); MaSp2 (Lh), L. hesperus major ampullate spidroin 2, residues 1–151 (GenBank accession no. ABR68855); MaSp2 (Lg), L. geometricus major ampullate spidroin 2, residues 1–124 (GenBank accession no. ABY67417); MaSp1-like (Lg), L. geometricus major ampullate spidroin 1-like, residues 1–151 (GenBank accession no. AAZ15320, modified as in[25]); MaSp1 (Lg), L. geometricus major ampullate spidroin 1 variant 1 locus 2, residues 1–151 (GenBank accession no. ABY67428); MaSp1 (Lm), Latrodectus macta [file pone.0052293.s003.tif]

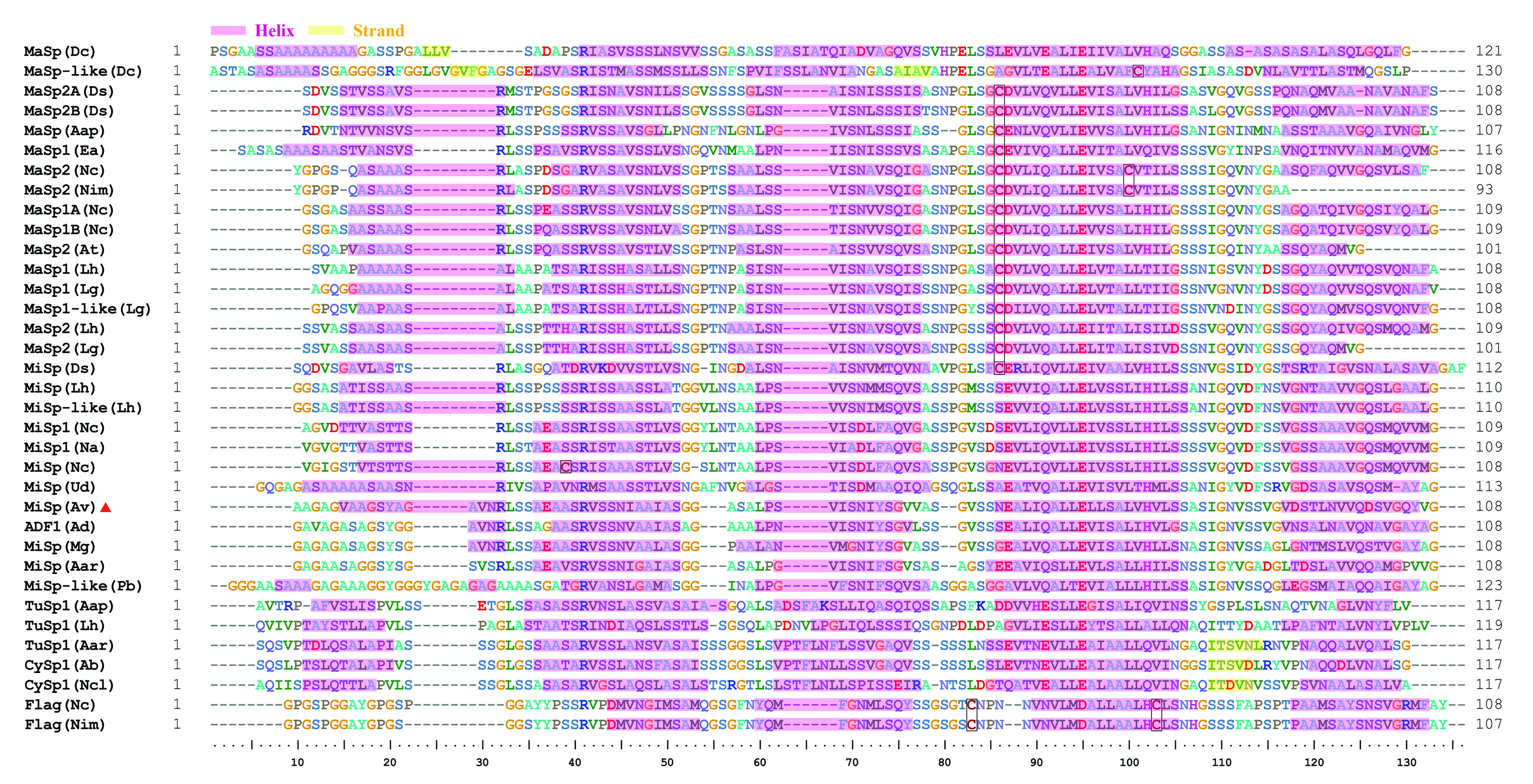

Supplement: Figure S4 — Sequence alignment of nonrepetitive C-terminal regions. The nonrepetitive C-terminal sequences are aligned by ClustalW2 using default parameters and amino acids are shown in different colors. α-helices and strands predicted with PSI-Pred v3.0 are shaded in red and yellow, respectively. The red triangle indicates A. ventricosus MiSp C-terminal sequence. Cysteines are in box. The sequences shown are the following: MaSp(Dc), D. canities major ampullate spidroin (GenBank accession no. ADM14316); MaSp-like(Dc), D. canities major ampullate spidroin-like protein (GenBank accession no. ADM14318); MaSp2A (Ds), D. spinosa major ampullate spidroin 2a (GenBank accession no. ABD61593); MaSp2B (Ds), D. spinosa major ampullate spidroin 2b (GenBank accession no. ABD61594); MaSp (Aap), A. aperta major ampullate spidroin (GenBank accession no. AAT08436); MaSp1(Ea), E. australis major ampullate spidroin 1 (GenBank accession no. CAJ00428); MaSp2 (Nc), N. clavipes major ampullate spidroin 2 (GenBank accession no. AAT75317); MaSp2 (Nim), N. inaurata madagascariensis major ampullate spidroin 2 (GenBank accession no. AF350278_1); MaSp1A (Nc), N. clavipes major ampullate spidroin 1 (GenBank accession no. AAT75312); MaSp1B (Nc), N. clavipes major ampullate spidroin 1 (GenBank accession no. AAT75311); MaSp2 (At), A. trifasciata major ampullate spidroin 2 (GenBank accession no. AAZ15372); MaSp1 (Lh), L. hesperus major ampullate spidroin 1 (GenBank accession no. ABR68856); MaSp1 (Lg), L. geometricus major ampullate spidroin 1 (GenBank accession no. AF350273_1); MaSp1-like (Lg), L. geometricus major ampullate spidroin 1-like (GenBank accession no. AAZ15321); MaSp2 (Lh), L. hesperus major ampullate spidroin 2 (GenBank accession no. ABR68855); MaSp2 (Lg), L. geometricus major ampullate spidroin 2 (GenBank accession no. AF350275_1); MiSp(Ds), D. spinosa minor ampullate spidroin (GenBank accession no. ABD61589); MiSp(Lh), L. hesperus minor ampullate spidroin (GenBank accession no. ADM14322); MiSp-li [file pone.0052293.s004.tif]

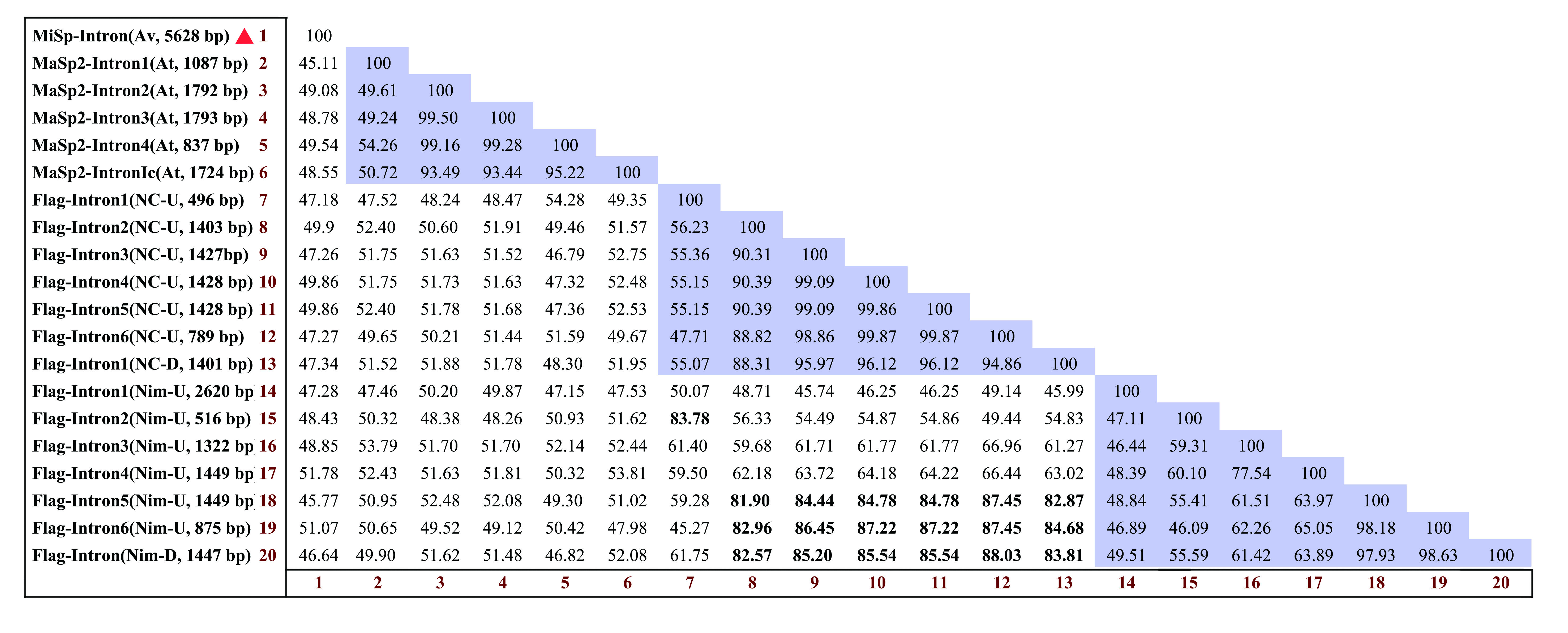

Supplement: Figure S5 — Percent pairwise sequence identities between introns from A. ventricosus MiSp, A. trifasciata MaSp2, and N. clavipes and N. inaurata madagascariensis Flag spider genes. The scores within the same gene sequences are shaded in blue. Scores over 80 are bold. The red triangle indicates A. ventricosus MiSp intron. The sequences shown are the following: MiSp-intron (Av) is from A. ventricosus minor ampullate spidroin gene sequence (GenBank accession no. JX513956); MaSp2-intron1∼4 (At) is from A. trifasciata major ampullate spidroin 2 gene (GenBank accession no. DQ059136S1); MaSp2-intronIc (At) is from A. trifasciata major ampullate spidroin 2 gene (GenBank accession no. DQ059136S2); Flag-intron1∼6(Nc-U) is from N. clavipes flagelliform silk protein (Flag) gene (GenBank accession no. AF218621S1); Flag-intron1 (Nc-D) is from N. clavipes flagelliform silk protein (Flag) gene (GenBank accession no. AF218621S2); Flag-intron1∼6(Nim-U) is from N. inaurata madagascariensis flagelliform silk protein (Flag) gene (GenBank accession no. AF218623S1); Flag-intron (Nim-D) is from N. inaurata madagascariensis flagelliform silk protein (Flag) gene (GenBank accession no. AF218623S2). (TIF) [file pone.0052293.s005.tif]
